# Accelerating Data Set Population for Training Machine Learning Potentials with Automated System Generation and Strategic Sampling

**DOI:** 10.1021/acs.jctc.5c00616

**Published:** 2025-07-02

**Authors:** Alberto Pacini, Mauro Ferrario, Maria Clelia Righi

**Affiliations:** † Department of Physics and Astronomy, 9296 University of Bologna, 40127 Bologna, Italy; ‡ Dipartimento di Scienze Fisiche, Informatiche e Matematiche, 9306 Università di Modena e Reggio Emilia, Via Campi 213/A, 41125 Modena, Italy

## Abstract

Machine Learning Interatomic Potentials (MLIPs) offer a powerful way to overcome the limitations of *ab initio* and classical molecular dynamics simulations. However, a major challenge is the generation of high-quality training data sets, which typically require extensive *ab initio* calculations and intensive user intervention. Here, we introduce Strategic Configuration Sampling (SCS), an active learning framework to construct compact and comprehensive data sets for MLIP training. SCS introduces the usage of *workflows for the automated generation and exploration of systems*, collections of MD simulations where geometries and run conditions are set up automatically based on high-level, user defined inputs. To explore nontrivial atomic environments, initial geometries can be assembled dynamically via *collaging* of structures harvested from preceding runs. Multiple *automated exploration workflows* can be run in parallel, each with its own resource budget according to the computational complexity of each system. Besides leveraging the MLIP models trained iteratively, SCS also incorporates pretrained models to steer the exploration MD, thereby eliminating the need for an initial data set. By integrating widely used software, SCS provides a fully open-source, automatic, active learning framework for the generation of data sets in a high-throughput fashion. Case studies demonstrate its versatility and effectiveness to accelerate the deployment of MLIP in diverse materials science applications.

## Graphical abstract

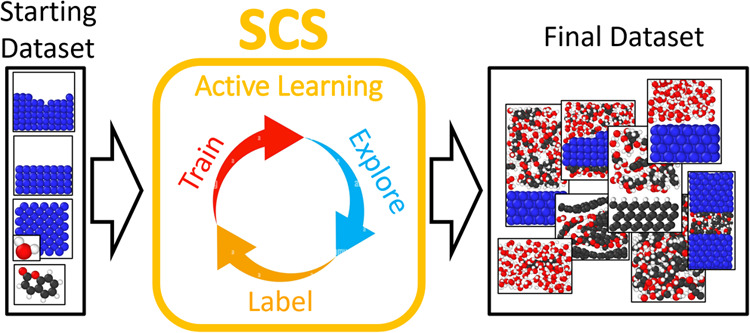

## 1 Introduction

Quantum mechanical *ab initio* simulations nowadays are essential tools for understanding the behavior of materials at microscopic level. However, these calculations are computationally expensive, limiting their application to small size systems and short time scales.^1^ Recently, machine learning interatomic potentials (MLIPs) have emerged as a promising solution to overcome these limitations.^2,3^ By learning from quantum mechanical data, MLIPs can reproduce atomic energies and forces at a fraction of the computational cost while maintaining near-*ab initio* accuracy. MLIP has extended the space and time scales accessible by simulations, paving the way to *in silico* nanoscale experiments across all domains of material science.^4–6^ MLIP decomposes the total energy of the system with a sum of local atomic energies which are inferred from their local atomic environments. Local environments are represented by descriptors, high-dimensional feature vectors encoding information on the neighborhood of each atom.^7,8^ During recent years most of the research has been focused on their development, giving rise to a plethora of atomic descriptors.^9–11^ The most important breakthrough was achieved by adopting equivariant descriptors, further propelled using graph neural networks and message-passing architectures.^12,13^ Charge-aware and long-range descriptors are additional research directions that are currently being actively explored.^14,15^ While much of the research is devoted to architecture design, much less is dedicated to aid data set creation, especially for complex system simulations. The possibility of deploying MLIPs in application- and system- specific tasks involves the need for comprehensive data sets containing thousands of expensive *ab initio* calculations. The creation of data sets is inefficient and prone to bias if performed manually. Standard methods such as *Ab Initio* Molecular Dynamics (AIMD) cannot efficiently produce uncorrelated and diversified atomic configurations in reasonable times. Recently, ready-to-use off-the-shelf models, so-called pretrained, universal or foundation models, have been made available.^16–19^ Although trained on vast available databases, they often lack the accuracy needed for simulating specific systems. Nonetheless, they offer an excellent starting point for fast and preliminary exploration of the chemical configuration space of interest. Active learning is another approach, coming from the realm of machine learning and data science. Active learning iteratively augments a data set by selectively querying the most informative data points. In the context of deep neural network, active learning techniques often rely on query-by-committee methods.^20^ Several active learning schemes for MLIPs have been proposed to accelerate data set constructions by selecting only relevant atomic configurations that are worth processing with expensive *ab initio* calculations.^21–24^ The method iteratively updates the data set following three main steps. In the first step, multiple MLIP models are trained. The models share the same architecture, but their weights or data sets are randomized at the beginning of the training. In the second step one of the models is used in the MD simulation that explores the atomic configuration space. The other models in the ensemble are instead used to calculate the “disagreement” on some physical observables, usually energies and forces, along the trajectories produced by MD. Quantifying the disagreement between the models, often called uncertainty quantification (UQ), lies at the core of query-by-committee methods. UQ is used as a quantitative measurement of confidence in the prediction on new data.^25^ In the last step, atomic configurations associated with uncertainty higher than a given threshold are first selected and then computed using *ab initio* methods obtaining quantum accurate values of the observables. The new data are added to the initial data set completing the active learning loop, and the whole procedure can be iterated until the uncertainty on new configurations becomes sufficiently low.

Current active-learning frameworks typically relies on sampling strategies such as standard molecular dynamics or more elaborate simulation schemes.^26^ However, users must still prepare initial geometries and simulation conditions—a time-consuming process that relies on advance knowledge of the local atomic environments likely to occur in large-scale simulations. Such prior assumptions can introduce bias, particularly for heterogeneous systems (interfaces, molecular mixtures,...) and may fail to capture unexpected, nontrivial configurations that emerge during extended runs. Conventional *ab initio*-sized simulations, with geometries fixed *à priori*, are not suited to uncover these hidden complexities. To overcome these limitations, we introduce Strategic Configuration Sampling (SCS), an open-source, active-learning framework that automatically generates comprehensive, uncorrelated, and compact data sets tailored to system-specific MLIP for large-scale simulations. SCS introduces *workflows for the automated generation and exploration of systems*—collections of MD simulations (phases) whose geometries and run conditions are defined by a simple, high-level syntax. During each phase, system geometries are assembled automatically from user-provided inputs and dynamically via “collaging,” i.e., by stitching together structures harvested from previous phases. The same syntax enables systematic screening of structural combinations and the application of diverse MD conditions, minimizing manual intervention and enabling a high-throughput exploration of complex dynamical events. The simulations in exploration phases are executed either via the LAMMPS^27^ or the ASE^28^ packages, while geometry initialization is performed with the optional integration of the Packmol software.^29^ The simulation in each phase generates a trajectory of atomic configurations which is then uniformly sampled over time using a query-by-committee approach. The sampled data are computed with *ab initio* single point DFT calculations throughout the SCS’s Quantum Espresso interface.^30^ SCS currently supports two popular open-source MLIP software, enabling users to train ensemble of MACE^13^ or DeePMD-kit^31^ models. Users must supply the native input files for their chosen MLIP type, and the SCS automated active learning procedure then populates the training data set proceeding within the chosen MLIP framework. In future releases this could eventually be circumvented introducing unified training and inference frameworks, that abstracts across distinct MLIP architectures, using approaches such as the one recently introduced with the work of Deep-GNN.^32^ Indeed, given the rapidly evolving field of machine-learning-driven atomistic simulation, it will be more and more paramount to incorporate timely advances in enhancing MLIP interoperability for both end users and developers across the many distinct MLIP architectures.

## 2 Methods

### 2.1 SCS Workflow Structure

The high-level structure of SCS, depicted on the left of Figure 1, is similar to other active learning frameworks.^22–24^ Each iteration begins with an ensemble of models—identical in architecture but initialized with different random seeds—trained on the current data set. The user can choose to train MACE or DeePMD-kit ensemble of models. These two architectures are somewhat complementary: DeePMD-based models are computationally efficient thus ideal for exploration of large systems on long time-scales,^33,34^ meanwhile MACE-based models emphasize data accuracy and generalization due to their robust equivariant-message passing architecture.^35,36^ Once trained, the ensemble is used to perform exploration by MD, targeting relevant regions of the configuration space. One model in the ensemble pilots MD simulations, while the others allow the computation of deviations, producing trajectories of uncertainties on the atomic forces. SCS focuses on these MD explorations to visit nontrivial atomic configurations which can enrich the data sets with significant and diverse chemical environments. This is achieved through *workflows for the automated generation and exploration of systems*, pipelines of high-level, user-defined ML-driven simulations (phases). Within each phase, the user defines Geometry and Run conditions. Geometries are assembled automatically based on user request by combining atomic structures in several ways: from ASE-readable files, using Packmol and by selecting output structures from earlier phases. This last feature, called *collaging*, is a dynamical way of assembling complex geometries and it is sketched on the inset in Figure 1. Phase0 and Phase1 control the simulations of 100 and 110 diamond interfaces in environmental conditions containing water and oxygen molecules. Their initial geometries are set up using a combination of user-defined files (for surfaces) and Packmol (for molecules). These MLIP-driven simulations explore surface passivation reactions, producing hydroxyl- and oxygen- terminated surfaces. Phase2 employs *collaging* to carve out the resulting reconstructed and passivated diamond surfaces and stitches them with intercalated glycerol molecules randomized by Packmol. This process realizes a complex interfacial system that is ready to undergo subsequent exploration subjected to temperature and load conditions. The modular design of the *exploration workflows* enables seamless and automatic navigation of such diverse chemical environments. More examples are presented in Section 3.

**Figure 1:**
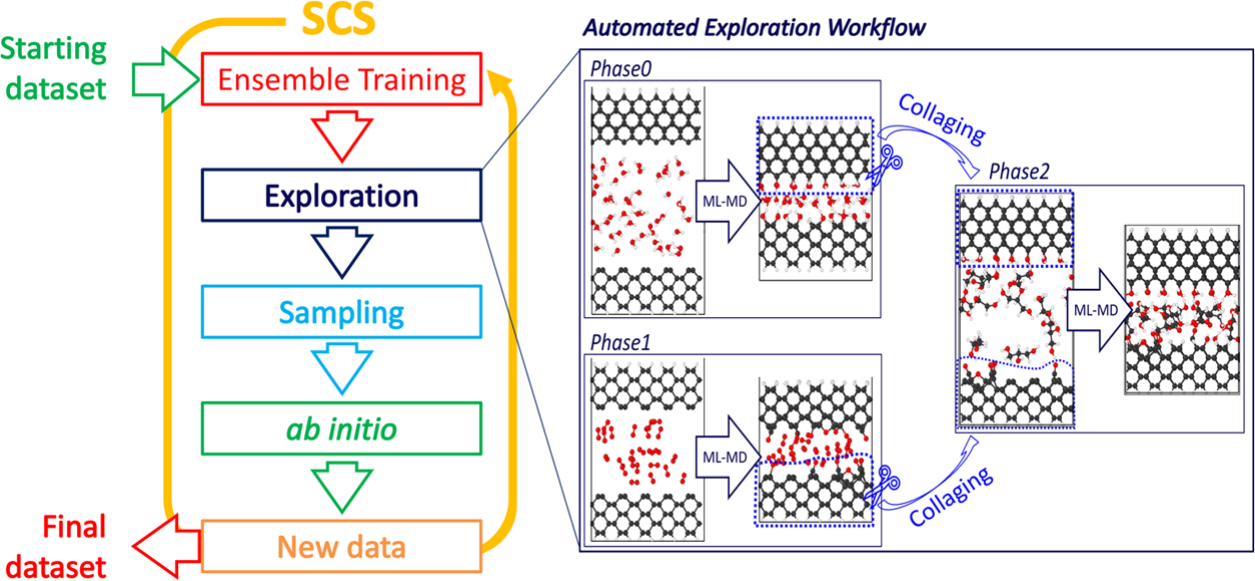
Schematic of SCS active learning loop. SCS iteratively updates an initial data set by sampling new configurations using a query-by-committee scheme and computes them with *ab initio* method. SCS introduces *workflows for the automated generation and exploration of systems* such as “playground” for the MLIP ensemble under the user supervision. These *workflows* define multiple exploration phases with interdependent geometries through “*collaging*”, realizing the automatic exploration of complex atomic environments.

The exploration MD of each phase is followed by the sampling process where the per-component force deviation is computed as the standard deviation of the ensemble

1 $$\sigma^{2}\left(F_{i,\alpha }\right)=\frac{1}{M}\sum_{j=1}^{M}{\left(F_{i,\alpha }^{j}-\left\langle F_{i,\alpha }\right\rangle \right)}^{2}$$

σis defined to be the ensemble uncertainty on *F*_*i*,α_, the Cartesian component α of the force acting on atom *i*. The term $\left\langle F_{i,\alpha }\right\rangle =\frac{1}{M}\sum_{j}^{M}F_{i,\alpha }^{\left(j\right)}$ is the ensemble average of *F*_*i*,α_, for an ensemble constituted by *M* models, i.e., *M* neural networks trained with the same data set but initialized with different random seed. SCS scans the trajectories produced by the *exploration workflows* and collects the frames with high uncertainties. Since larger forces have naturally larger uncertainties, each uncertainty can be normalized by the magnitude of the atomic force. The frame with highest uncertainty (typically σ/(|⟨*F*_*i*,α_⟩| + ϵ) ≥ 1 where ϵ filters out near zero forces) is harvested from equal-time windows along the trajectory, ensuring the selected frames are well spaced in time. This uncorrelation step decreases the redundancy in the data set while reducing the number of samples to be computed. Selected frames undergo single-point DFT calculations (scf) via SCS’s Quantum ESPRESSO interface. Crucially, each system’s scf jobs can be independently configured, enabling efficient resource allocation. Moreover, SCS checks that the sampled frames have no overlapping atoms to avoid unphysical configurations that would lead to convergence failures. After the *ab initio* calculations, SCS filters out configurations with extreme atomic forces (default is 30 eV/Å) to prevent training instabilities due to force outliers. Cleaned, high-quality data are then added to the training set, completing the active learning loop. The first iteration of active-learning requires an initial data set to train the MLIP ensemble. While users often already possess *ab initio* data for their systems of interest, SCS also integrates the usage of pretrained or foundation (universal) models at any stage of the exploration MD. This flexibility is particularly valuable when no suitable initial data set exists or when existing data are insufficient to yield stable ML-driven dynamics (see Section 3.2).

## 3 SCS Usage

This section showcases several SCS sessions to present the characteristic features of the software. Foremost among these are SCS’s exploration workflows–modular pipelines that dynamically assemble sophisticated geometries, via *collaging* of previous outputs as well as using user defined structures, and execute complex dynamical simulations, showcasing exceptional flexibility across diverse systems and conditions.

### 3.1 Basic Exploration Workflow

The main motivation behind *automated exploration workflows* is to efficiently explore nontrivial configuration space. Moreover, their simple syntax also offers an easy interface when dealing with more basic systems and conditions. We show a basic example of such a *workflow* by focusing on Diamond-like-Carbon (DLC) systems. DLC is a class of amorphous carbon material that finds numerous applications in antiwear coatings for tooling machinery, engines and biomedical materials.^37–39^ Amorphous carbon consists of a mixture of sp^2^ and sp^3^-bonded carbon atoms depending on the value of the density. sp^3^-Rich DLC occurs when the density is closer to diamond density, sp^2^-rich DLC when it is closer to graphite density.^40^ The goal is to collect a data set of atomic configurations for DLC systems. Graphite and diamond are chosen as starting points for the *automated exploration workflow*; their equilibrium structure will undergo melt-quench dynamics to generate the relevant local environments for amorphous DLC. An example of such simple *workflow*’s input is shown in Figure 2. At variance with other active learning frameworks that would require the user to intervene at each stage, SCS automatically executes each phase of the *workflow* sequentially. Phase0 will use the current MLIP model to relax the system geometry and no sampling will be performed. This phase has the purpose of preparing the system for the subsequent exploration phases. Phase1 specifies an annealing dynamic where the temperature is steadily increased from 1 to 9000 K over a 1 ns-long run, while Phase2 simulates the quenching process where the liquid diamond is cooled down to room temperature. Figure 3 shows the uncertainty of the MLIP ensemble along the dynamics in blue, while the red bars indicate times at which the configurations are sampled. During the first iterations (top panel) the MLIP ensemble is unstable, the uncertainty skyrockets and for temperatures above 4000 K the carbon atoms are attracted toward unphysical-short distance configurations (insert B). These configurations are erroneously predicted to be stable, and persist throughout the simulation time, preventing further sampling. SCS sampling policy enriches the data set with time-uncorrelated high uncertainty configurations while still checking that they do not contain overlapping atoms to avoid nonconverging *ab initio* calculations. At iteration 6, after ∼500 new collected DFT configurations, the MLIP knowledge has advanced enough, and the ensemble is now stable throughout the entirety of the melt-quench dynamics. This ensures that new configuration space regions can now be sampled properly, including the liquid carbon phase (C) with recrystallization events (D) possibly occurring during the quenching phase.

**Figure 2:**
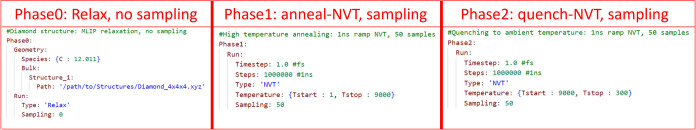
Input file for SCS’s *exploration workflow*. This yaml file is required for each system that needs to be explored. The input is divided into “Phase blocks” containing a Geometry and a Run section. When Geometry is not specified it is initialized from the final geometry of the previous phase.

**Figure 3:**
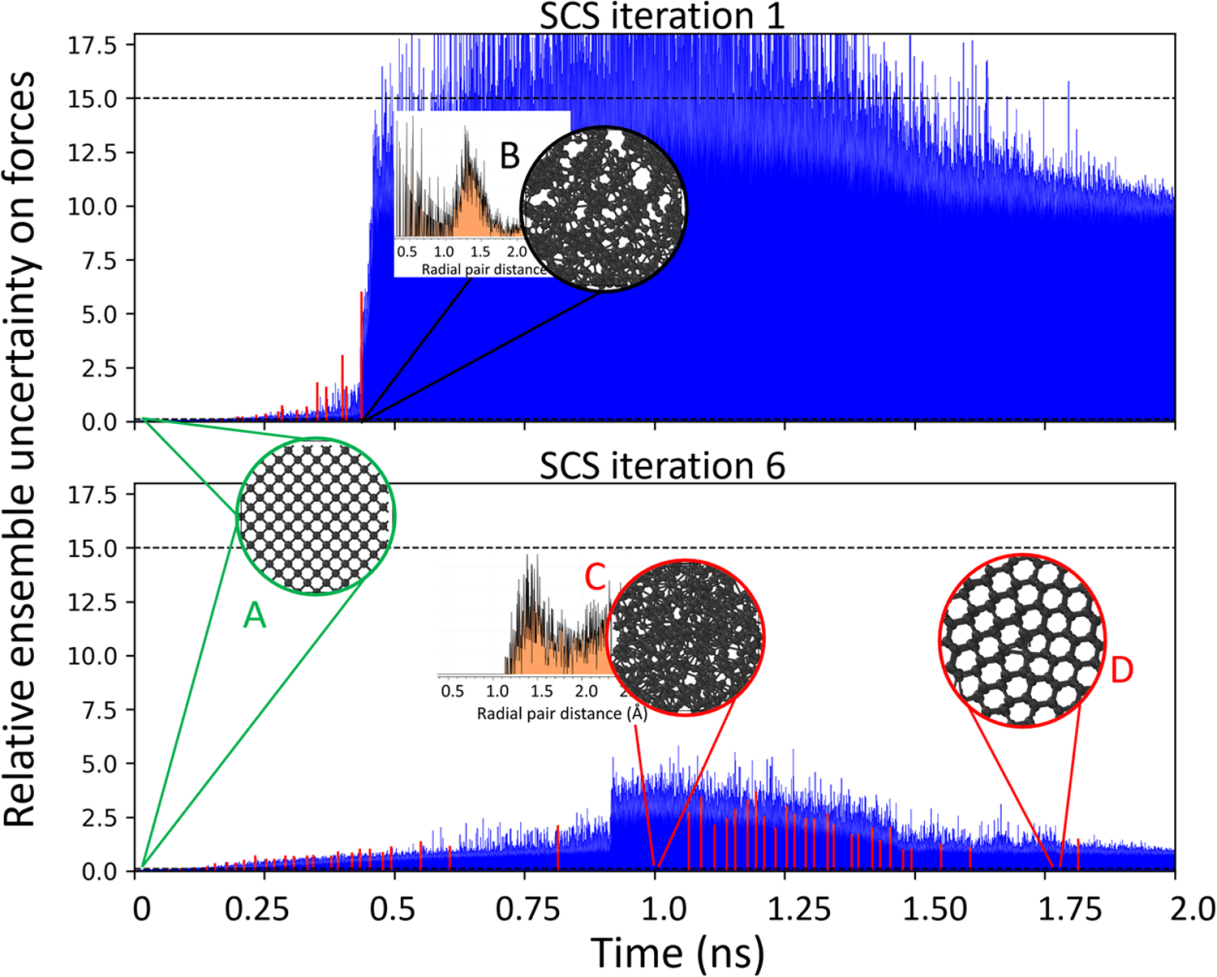
Learning process during active learning iterations of the melt-quench process for a diamond bulk (A). Blue lines shows the ensemble uncertainty for each atomic configurations while red lines highlight only the selected configurations. During the first iterations the MLIP is unstable, predicting unphysical-short distance atomic geometries that are not sampled by SCS to avoid *ab initio* convergence issues (B). After 6 SCS iterations the ensemble can reproduce a stable liquid carbon phase (C) that recrystallizes during the 1 ns long quenching process (D).

### 3.2 Boosting Exploration with Pretrained Models

Having an initial data set is crucial to supply the MLIP ensemble with some knowledge of the relevant configuration space. If the initial data set is too small and lacks proper sampling of atomic energy barriers, the MLIP will struggle to generate meaningful, physical structures that have some hope of converging when computed *ab initio*. This “exploration bottleneck” afflicts the first six iterations of active learning shown previously, forcing the user to wait until the required MLIP stability is reached. The recent deployments of so-called universal or pretrained models can be used to overcome this exploration bottleneck.^17,18^ Universal models are typically large models with many parameters, trained on vast existing data sets.^41,42^ Although these pretrained models are usually slower and often lack the accuracy for targeting specific systems and conditions, they are extremely valuable in exploring the high energy landscape necessary to stabilize system specific-MLIP. SCS seamlessly integrates the usage of pretrained models that can be employed to pilot explorative MD simulations at any stage of the *automated exploration workflow*. Figure 4 shows the speed-up gained in the learning progress for the same DLC application discussed above. During the first iteration the universal model drives the anneal-quench exploration enabling the sampling up to very high temperatures. At the beginning of iteration 2, the MLIP ensemble is trained from scratch on these collected data (∼90 frames), representing the initial data set. The trained ensemble turns out to be stable enough to complete the whole melt-quench dynamics which can be sampled entirely already at the second iteration. The seamless interface with pretrained or universal models is a novel feature that permits and accelerates the active learning when a proper initial data set is lacking.

**Figure 4:**
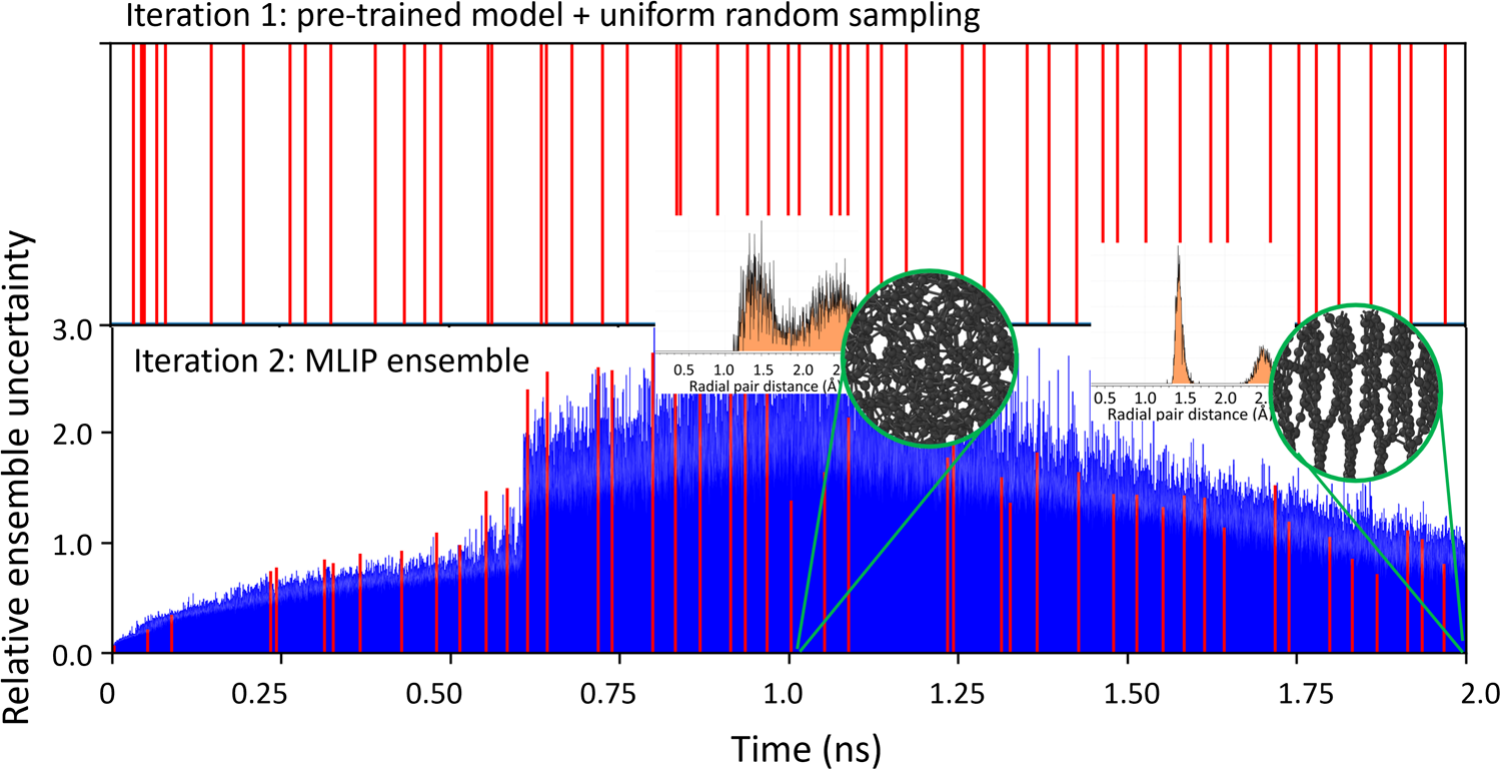
Learning progress for the same DLC systems. During the first iteration the exploration dynamics are driven by an external universal model (mace-omat-medium). Blue lines shows the ensemble uncertainty for each atomic configurations, while red lines highlight only the selected ones. Since there is no ensemble, this trajectory is uniformly sampled and provides the initial data set for the MLIP ensemble. With as few as 90 sampled configurations, the MLIPs trained at iteration 2 can already explore the whole melt-quench process without instabilities.

### 3.3 Parallel Exploration of Different Systems

In this section we discuss the feature of heterogeneous exploration, presenting an SCS session which has been used in the application to methane pyrolysis on iron nanoparticles. The study of hydrogen production mechanisms is crucial to achieve net zero carbon emission and methane pyrolysis is one of the core technologies involved in producing low-cost, low-emission hydrogen.^43–46^ The prototypical large-scale ML-driven simulation includes an iron nanoparticle inside a methane atmosphere as depicted on the left panel of Figure 5. Since the MLIP decomposes the total energy as a sum of local atomic energies, it is important to create a data set comprehensive of all the local atomic environments. The colored framed boxes illustrate a simple subdivision where each subsystem contains the relevant local environments for the large-scale simulation. The boxes allude to cell sizes sufficiently small to permit agile *ab initio* calculations. SCS allows the user to independently explore several systems simultaneously using the same MLIP ensemble. In this example four systems are set up within the same SCS session, each explored by its own *automated exploration workflow*. Systems remain independent also during the *ab initio* data generation step, allowing the user to allocate distinct resources tailoring system-specific computational needs for parallelization and convergence control. Differently from other active learning frameworks, this approach significantly accelerates the data generation process for heterogeneous systems, allowing to prioritize resource-intensive systems–like those involving iron surfaces– optimizing overall computational efficiency and enabling strategic resource distribution.

**Figure 5:**
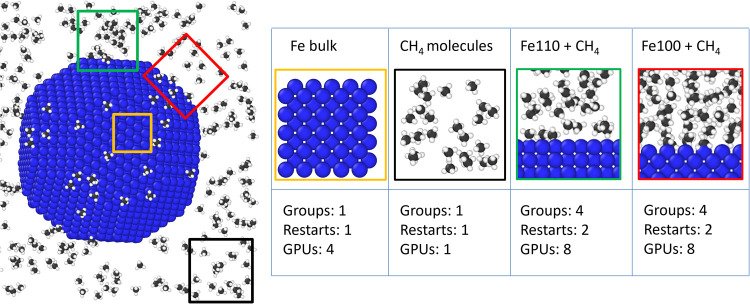
Multiple *automated exploration workflows* are carrying on in parallel, one for each system in colored frames. The systems are treated independently also during the *ab initio* computations, so that distinct computational resources can be assigned according to each system’ s computational complexity.

### 3.4 Automated System Generation

This paragraph highlights the novel mechanisms behind the automatic generation of system geometries and MD conditions realized by SCS’s *automated exploration workflows*. While maintaining a simple user-friendly syntax, such *workflows* automatize the construction of complex simulation scenarios, achieving the exploration of nontrivial atomic environments. This new approach resembles an high-throughput scheme applied to dynamical events, representing a leap forward toward the automatic construction of data set for MLIPs.

#### 3.4.1 Automatic Generation of System Replicas for Different Working Conditions

This section shows how SCS can deal with systems that require the coexistence of multiple, often indefinite, combinations of compounds or structures, such as complex surfaces, nanoparticles and molecular mixtures. One example of such application was applied to develop a data set for complex lubricant mixtures commonly used for tribological applications.^47^ In such cases, the large-scale molecular mixture can have, in general, undefined local concentrations and the MLIP needs to have knowledge of these distinct local environments. One prototypical situation has been depicted in Figure 6 where the system is composed of a mixture of five distinct compounds. The combinatorial space of possible local environments is vast and there is little hope of exploring them using the same few initialized geometries. For every phase of the *automated exploration workflow*, the user can specify a list of distinct compounds together with their relative concentrations, or a target density. SCS will automatically create geometries that satisfy user requirements by randomly placing molecules and shuffling their relative concentrations. This feature is enabled through SCS’s Packmol interface and it can be used to efficiently and effortlessly screen throughout the configurational space of the mixture. The same procedure can be applied for bulk and surface structures: at each exploration phase one single structure will be randomly selected from the pool of structures defined by the user creating randomized configurations that enriches the data set in a completely automatized way.

**Figure 6:**
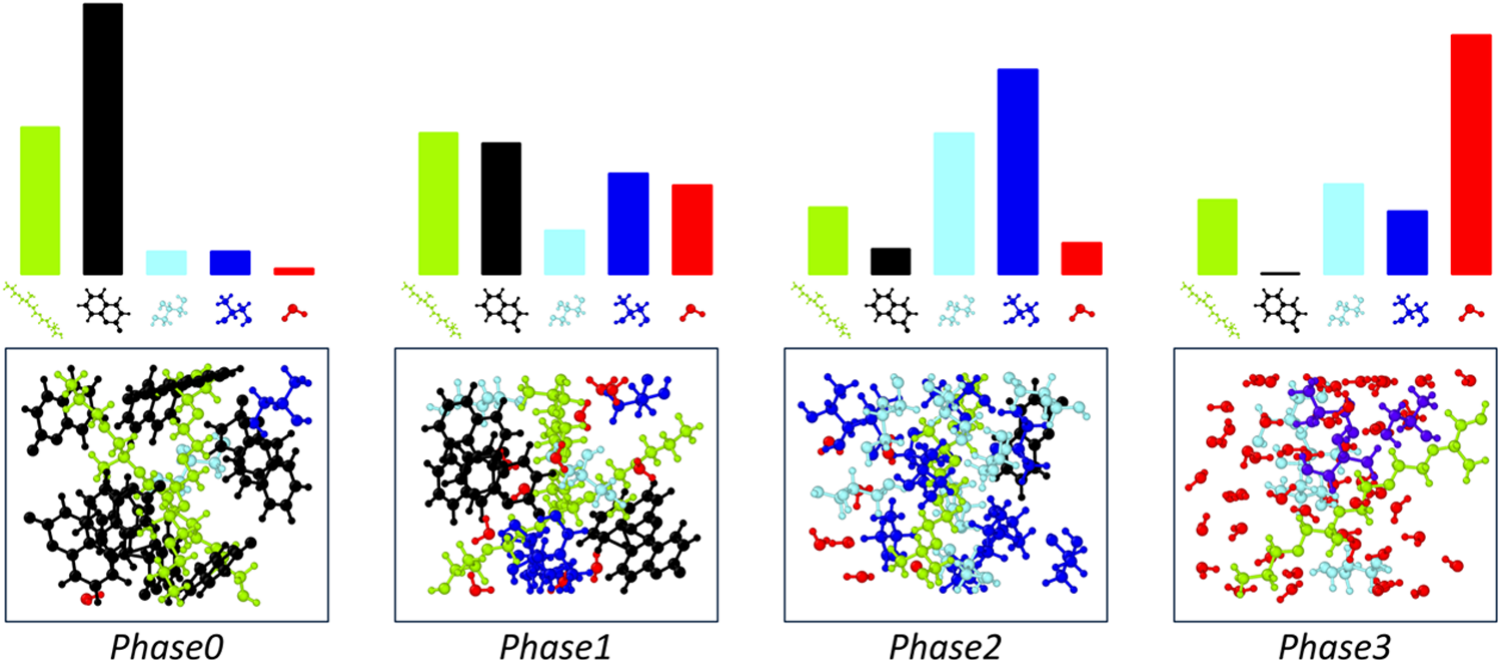
Subsequent phases of an *automated exploration workflow* applied to molecular mixtures. The user can define multiple compounds to be placed inside *ab initio*-sized cell with a specific target density. SCS automatically shuffles molecules’ positions and screens different possible molecular concentrations, maximizing the diversity among the chemical environments.

#### 3.4.2 Automatic Generation of Complex Systems by Collaging

In Section 2 we anticipated that SCS relies on *automated exploration workflows* to create complex systems. Figure 7 shows an example of such *workflow* able to capture nontrivial atomic environments coming from the results of complex dynamical simulations. Similar SCS *workflows* have been used to develop a data set applicable to the simulation of wear at the silica-diamond interface. The wear processes at the silica-diamond interface had already been investigated using *ab initio* methods^48,49^ and the usage of large-scale MLIP-driven simulations can provide further microscopic insights and statistical meaningfulness. The bottom-left corner of the image shows the few initial structures prepared in advance: H_2_O molecule, SiO2 molecule and the (110)-diamond surface. The *workflow* starts by randomly arranging several SiO_2_ molecules and annealing them to generate the amorphous silica with the user defined target density of 2.2 g/cm^3^. A box of water molecules is analogously generated, and it is glued to the brand-new amorphous silica bulk using the collaging feature. This new geometry is further annealed to explore the hydroxyl passivation events of the silica slab (Phase1). Collaging is then applied again to assemble the OH-terminated silica and the 110-diamond slab, forming an interface. The interface is first subjected to a loading force that welds the surfaces together (Phase2), then a pulling force progressively separates the two surfaces apart (Phase3), revealing valuable local environments for the large-scale wear simulations. *Automated exploration workflows* are iterated, representing an automatic “playground” for the MLIP ensemble in view of the large-scale simulations. The ease to set up such sophisticated *workflows* are the main workhorse of SCS which make it a framework suited for automatizing the active learning across different scenarios.

**Figure 7:**
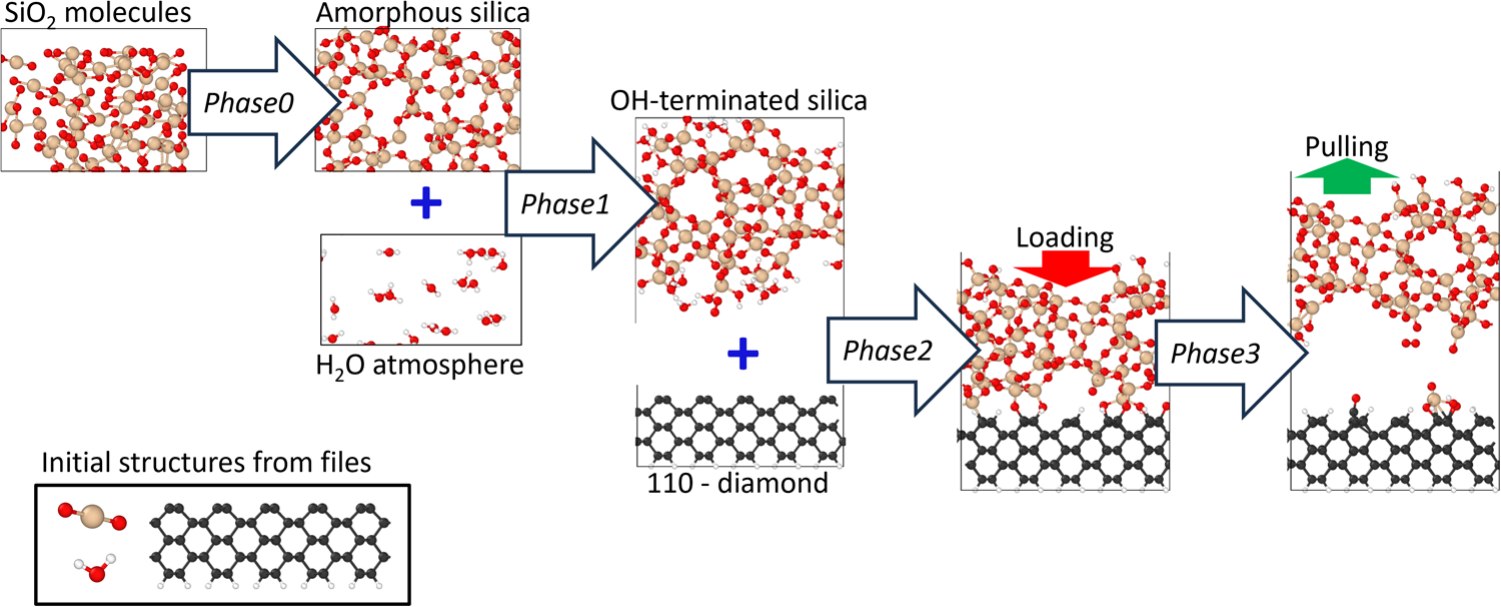
Complex *automated exploration workflow* for silica-diamond wear applications. Starting from simple geometries, SCS can generate sophisticated systems throughout the workflow’s phases: amorphous silica (Phase0), hydroxylated-silica (Phase1), silica-diamond interface under load (Phase2) and wear (Phase3). The blue addition symbols indicate the usage of the “collaging” feature to stich different structures.

## 4 Summary

In this work we present Strategic Configuration Sampling (SCS), a fully open-source active learning framework intended to automatize the creation of data sets suited for the deployment of MLIP in large-scale MD simulations of complex systems and processes. SCS innovates standard well-known active learning schemes, by introducing *workflows for the automated generation and exploration of systems*. These form collections of exploration MD simulations where initial geometries are automatically and dynamically assembled following a simple-syntax, user-defined input. The dynamic composition of system geometry by “*collaging*” the output structures from previous MD, permits the realization of nontrivial scenarios emerging as the result of possible dynamical events. This feature together with the automatization of system compositions and the screening of MD conditions allows the exploration of complex atomic environments while minimizing user intervention. Multiple systems can be explored in parallel by means of distinct *workflows* while assigning independent computational resources to execute their *ab initio* computations. Following the recent advancements in the world of MLIP, SCS integrates the use of pretrained or universal model to drive the MD simulations inside the *automated exploration workflows*. This feature guarantees a faster active learning framework in situation of data set deficiency. Overall, the novelty of *automated exploration workflows* lies in being an automatic “playgrounds” for the MLIP ensemble that, under the user supervision, approaches an high-throughput scheme for automatizing data sets population.

## Data Availability

Source code and data are available at the group’s repository: https://gitlab.com/triboteam/SCS.
